# Healthcare Professionals’ Resilience During the COVID-19 and Organizational Factors That Improve Individual Resilience: A Mixed-Method Study

**DOI:** 10.1177/11786329231198991

**Published:** 2023-09-19

**Authors:** Raquel Simões de Almeida, Ana Costa, Inês Teixeira, Maria João Trigueiro, Artemisa Rocha Dores, António Marques

**Affiliations:** 1 LabRP-CIR, ESS, Polytechnic University of Porto, Porto, Portugal; 2 Laboratory of Neuropsychophysiology, Faculty of Psychology and Education Sciences, University of Porto, Porto, Portugal

**Keywords:** Resilience, healthcare services, Covid19, healthcare professionals

## Abstract

Healthcare workers are a susceptible population to be psychologically affected during health crises, such as the recent COVID-19 pandemic. Resilience has been pointed out in the literature as a possible protective factor against psychological distress in crisis situations. This can be influenced by internal and external factors, such as individual characteristics and organizational factors. Thus, this study aims to characterize the overall resilience levels among healthcare professionals in Portugal and to understand the perspectives of this healthcare workers regarding organizational factors that improve individual resilience. This is a mixed-method study: a first quantitative study using a cross-sectional design to administer the Resilience Scale for Adults (RSA) to 271 healthcare professionals (Mage 33.90, SD = 9.59 years, 90.80% female), followed by a qualitative study through 10 in-depth interviews. The mean score for the total RSA was 178.17 (SD = 22.44) out of a total of 231. Qualitative analysis showed 4 major themes on factors that enhance resilience: “Professional’s Training,” “Support and Wellbeing Measures,” “Reorganization of Services” and “Professional Acknowledgment.” The findings may contribute to the development of targeted interventions and support systems to enhance resilience and well-being among healthcare workers.

## Background

The COVID-19 pandemic began in 2020 and since its onset millions of lives have been lost, public health systems have been in shock, and various economic and social disruptions have arisen.^1-4^ Thus, the pandemic seems to have significantly affected the mental health, well-being, and possibly work effectiveness of healthcare workers because they are generally at the forefront of a response to these adverse events.^5^ These findings can be explained by the occupational context of healthcare professionals that requires them to face several challenges in addition to those faced by the general population, such as fear and general uncertainty.^5-7^ Indeed, healthcare professionals are prone to direct exposure to pathogens, lack of or inadequate personal protective equipment, increased workload, burnout, or ethical issues related to decision making related to the well-being of their patients. In addition to these challenges, organizational issues such as the reorganization of workspaces and routines, or personal issues such as feelings of failure in the face of unfavorable prognoses or the lack of support caused, for example, by increased periods of separation from the family, contribute to an aggravation of the difficulties experienced by healthcare professionals.^7-9^

Building resilience in healthcare workers can protect them against negative job-related outcomes such as burnout, anxiety, and depression, and, as a result, enhance patient outcomes.^10-12^ Briefly, resilience can be defined as the ability to overcome adversity.^13^ However, Connor and Davidson^14^ believe that resilience is not a process, but rather a personality trait that encompasses a set of characteristics that enable individuals to cope with adversity and adapt to the circumstances. According to other authors, resilience may be influenced by internal and external factors,^15^ from individual characteristics, such as intelligence and problem-solving abilities, to contextual variables, namely family context, social context, and cultural and normative particularities.^16^

Studies have been conducted on the development of resilience, focusing on healthcare professionals.^17^ There are protective factors that enhance resilience and improve adaptation to high stress and burnout situations in healthcare professionals.^18^ These include the provision of literacy and training, interventions to create a sense of readiness, psychosocial support and treatment, monitoring the health status of healthcare workers, paying attention to the type of tasks, the mix of tasks and responsibilities, and the intensity and burden of those tasks, as well as working patterns and conditions.^17,19^ These and other organizational factors seem to contribute to increased individual resilience of healthcare professionals, which may also lead to improved quality of health services.

Given the data mentioned above, there is a need to focus research on resilience and recommendations to promote it, providing organizations and healthcare professionals with the tools to cope more effectively with adverse situations in the future. Thus, this study aims, based on the experiences of the COVID-19 pandemic, to characterize the overall resilience levels among healthcare professionals in Portugal and to understand the perspectives of Portuguese healthcare workers regarding the organizational factors that improve individual resilience. The specific objectives are: (1) to assess the overall resilience levels among healthcare professionals in Portugal, (2) to examine the relationship between resilience and demographic factors (eg, age, gender, professional role, years of experience), (3) to explore the association between resilience and workplace factors (eg, number of working hours per week, overall health rating), (4) to understand the mechanisms used by healthcare professionals to enhance their resilience and deal with the pandemic, and (5) to provide strategic recommendations for improving healthcare services.

## Methods

### Design

This study is part of the European project “Empower4Pandemias—Learning from Covid19.” Briefly, the main objective of the project is to pilot a blended learning training to enhance the resilience skills of professionals and decision-makers in the health and emergency sector and related public administrations to strengthen their individual and organizational resilience to better cope with a pandemic crisis.

This is an explanatory sequential mixed method study that combines quantitative and qualitative methodologies.^20^ In this design, qualitative data (interviews) supplement quantitative data (questionnaires) to create a deeper understanding of factors that improve individual resilience. The study started as a quantitative study, with a cross-sectional design, administered the Resilience Scale for Adults (RSA) to identify the level of resilience of healthcare professionals. Then, a qualitative design was followed based on in-depth interviews to explore the organizational factors associated with their individual resilience. The quantitative phase was held between April and May 2023 and the qualitative phase was conducted between May to June 2023.

### Participants

The study participants for the 2 stages were healthcare professionals (ie, doctors, nurses, allied health professionals and psychologists). The inclusion criteria outlined for this study were having worked in healthcare services during the COVID-19 pandemic in Portugal. For the survey, the sample number was deemed sufficient based on the sample calculation formula^21^ which showed that for a total of114 000 health professionals, the sample size should be 246. In stage 2, participants were recruited through the non-probability purposeful sampling method which aimed to select individuals with the highest and lowest resilience scores in the previous survey.

### Materials

The questionnaire was composed of questions reviewed by experts and was organized into 3 sections: (1) sociodemographic data, (2) work-related issues during the COVID-19 pandemic, (3) Resilience Scale for Adults.

The Resilience Scale for Adults (RSA)^22^ is a self-administered questionnaire that assesses several characteristics of resilience, containing items that in their original structure are organized into the following 6 factors: Perception of Self (PoS), Planning for the Future (PF), Social Skills (SS), Family Cohesion (FC), Social Resources (SR), and Structured Style (StS). This scale can be applied to adults in any context. Regarding the total score of the scale, each question is rated on a Likert scale, ranging from 1 to 7, with higher scores meaning higher levels of resilience. There are 17 items that should be inverted. To obtain the score for each factor 1 should calculate its average or use the total value. This scale is validated for the Portuguese population and has a very good internal consistency with a Cronbach alpha of .94.^23^

The interview guide (see Supplemental Material) consisted of open-ended questions related to key areas of interest about the healthcare workers and lived experience during the pandemic. The interview was divided into 7 sections: (1) General questions about roles and responsibilities, (2) Experiences about the initial phase of the pandemic, (3) Work routines and challenges during the COVID-19 pandemic, (4) Patient care during the pandemic, (5) Organizational support, and (6) Individual skills and stress.

### Procedures

The characterization of the levels of resilience in the population and the collection of the variables under study were carried out through an online survey. Data collection was implemented via the Web-based survey platform Microsoft Forms platform. Meanwhile, in the second stage, data collection was carried out through in-depth interviews conducted by the researchers. The semi-structured interview guide was developed based on key literature and pilot-tested for clarity and completeness after the interview script was reviewed and approved by a panel of experts in this field. The interviews were conducted via Zoom by 2 researchers who were trained on how to conduct the interviews. The interviews lasted approximately 60 minutes. All interviews took place ensuring privacy, with no one else present apart from the participant and the interviewer. Interviews were audio-recorded with participants’ permission and professionally transcribed verbatim. Fieldnotes were written after each interview to record aspects of the interview that may not be captured on the recording such as general observations and thoughts. All participants provided their consent upon their involvement in the study. Ethical approval was sought from the ESS, Polytechnic University of Porto Ethics Committee (n. CE0111C).

### Data management and analysis

For quantitative study, descriptive analyses were used to characterize the sample, the result of the RSA and the 6 subscales. Pearson’s correlation coefficient was used to assess the association between total scale and subscales values and age, years of working as a healthcare professional, number of working hours per week, and how they perceived their health during the COVID-19. Comparisons of resilience total scale and subscales were performed using independent *t*-student and 1-factor ANOVA tests. All analyses were performed using the Statistical Package for the Social Sciences (SPSS) version 27.0 for Windows and a .05 alpha.

For qualitative study, verbatim transcriptions were made based on the interview recordings. Interview transcripts were analyzed using thematic analysis.^24^ Initially, a pre-analysis of the transcripts was performed through a free reading and relevant data were organized into meaningful codes. These codes were classified into potential resilience-related themes and their influence on health workers’ experiences during the COVID-19 pandemic. The emerging themes were reviewed by reading through all codes and the entire data set to confirm thematic validity and, after this validation, they were defined and named. The interviews, transcription, and thematic analysis were performed by the principal investigator and a research assistant, both occupational therapists. Results were discussed until a consensus on interpretation was reached. The codes and themes were reviewed by 2 other researchers prior to reporting. The thematic analysis was conducted using the software WebQDA.^25^

## Results

### Quantitative phase

Table 1 shows the sociodemographic characteristics of the 271 participants, with a mean age of 33.90 years old (SD = 9.59) and 246 (90.80%) were female. Almost 75.00% (n = 201) had a degree, 23.20% (*n* = 63) had a master’s degree, and 7 (2.60%) had a Ph.D. Most of the individuals were allied health professionals, 201 (74.20%), 43 (15.90%) were nurses and 10,00% (*v* = 27) were medical doctors or psychologists. During the COVID-19 period, participants worked mainly in secondary care institutions 103 (38.00%) or in long-term care 82 (30.30%), with 69 (25.50%) working in the private sector and only 16 (5.90%) in primary care settings and, in the same period, 92 (34.20%) worked shifts. The average quality of health of healthcare professionals during the pandemic was 6.47 in 10.

**Table 1.** Sociodemographic characteristics of the participants in the survey.

Variables		Frequency n (%)	M (SD)
Age	—-	—-	33.90 (9.59)
Quality of health during pandemic	—-	—-	6.47 (1.97)
Gender	Female	246 (90.80)	
	Male	25 (9.20)	
Education level	Bachelor’s degree	201 (74.20)	
	Master’s degree	63 (23.20)	
	Doctorate degree	7 (2.60)	
Occupation	Nurse	43 (15.90)	
	Allied health Profession	201(74.20)	
	Medical doctor/psychologist	27 (10.00)	
Organization	Primary care	16 (5.90)	
	Secondary care	103 (38.00)	
	Long-term care	82 (30.30)	
	Private sector	69 (25.50)	
Shift work (n = 269)	Yes	92 (34.20)	
	No	177 (65.80)	

Abbreviations: M, Mean; SD, standard deviation.

According to Table 2, the mean score obtained from the total RSA was 178.17 (SD = 22.44) out of a total of 231 possible points. The mean of subscales was 30.32 (SD = 9.95) out of 42 points to PoS, 20.52 (SD = 4.34) out of 28 points to PF, 31.27 (SD = 5.90) out of 42 points to SC, 20.36 (SD = 3.65) out of 28 points to PS, 33.40 (SD = 6.24) out of 42 points to FC, and 42.31 (SD = 5.44) out of 49 points to SS. Values in bold indicate significant *P* = 0.05

**Table 2.** RSA scale and subscales values according to participant sociodemographic characteristics.

	PoS		PF		SC		PS		FC		SS		Total RSA	
	M (SD)	*P*	M (SD)	*P*	M (SD)	*P*	M (SD)	*P*	M (SD)	*P*	M (SD)	*P*	M (SD)	*P*
Total participants	30.32 (9.95)		20.52 (4.34)		31.27 (5.90)		20.36 (3.65)		33.40 (6.24)		42.31 (5.44)		178.17 (22.44)	
Gender														
Female	30.24 (5.95)	.462	20.48 (4.34)	.661	31.13 (5.89)	.225	20.42 (3.70)	.360	33.68 (5.95)	.077	42.43 (5.51)	.252	178.39 (22.56)	.6
Male	31.16 (6.03)		20.88 (4.47)		32.64 (5.94)		19.72 (3.08)		30.56 (8.27)		41.12 (3.65)		176.08 (23.75)	
Education														
Bachelor’s degree	29.98 (5.94)	.103	19.98 (4.45)	**.002**	30.78 (6.04)	.125	20.15 (3.67)	.295	33.16 (6.04)	.092	42.01 (5.76)	.312	176.07 (22.88)	**.034**
Master’s degree	30.98 (5.93)		21.95 (3.52)		32.56 (5.18)		20.92 (3.68)		34.52 (5.87)		43.11 (4.25)		184.05 (20.06)	
PHD	34.29 (5.25)		22.86 (4.74)		34.00 (6.30)		21.14 (2.48)		29.71 (12.26)		43.57 (5.19)		185.57 (29.52)	
Occupation														
Nurse	28.81 (5.84)	**.007**	19.26 (4.73)	**.008**	28.86 (6.56)	**.011**	19.19 (3.94)	.071	33.19 (6.12)	.937	41.07 (6.26)	.264	170.37 (25.02)	**.018**
Allied Health	30.24 (5.90)		20.51 (4.28)		31.63 (5.71)		20.59 (3.53)		33.39 (6.27)		42.53 (5.26)		178.90 (21.81)	
Medical doctor/psychologist	33.33 (5.66)		22.56 (3.48)		32.44 (5.36)		20.52 (3.82)		33.74 (6.45)		42.67 (5.32)		185.26 (22.22)	
Organization														
Primary care	26.69 (5.10)	.883	19.69 (4.78)	.824	31.00 (4.68)	.357	20.81 (3.66)	.838	33.56 (6.36)	.821	42.50 (6.20)	.829	177.25 (17.32)	.837
Secondary care	30.58 (6.01)		20.61 (4.66)		30.47 (6.43)		20.14 (3.48)		32.90 (6.25)		41.90 (5.69)		176.58 (24.04)	
Long-term care	30.34 (5.97)		20.68 (4.17)		31.91 (5.58)		20.33 (3.93)		33.73 (5.97)		42.57 (4.84)		179.57 (21.35)	
Private sector	29.93 (6.02)		20.30 (3.10)		31.67 (5.62)		20.57 (3.60)		33.55 (6.57)		42.49 (5.63)		178.51 (22.95)	
Shift work														
Yes	30.65 (5.76)	.497	20.62 (4.72)	.766	30.93 (6.27)	.483	20.16 (3.40)	.588	33.73 (5.83)	.561	42.36 (5.39)	.997	178.46 (22.77)	.899
No	30.13 (6.08)		20.45 (4.17)		31.47 (5.72)		20.42 (3.78)		33.26 (6.47)		42.36 (5.47)		178.08 (22.75)	

Abbreviations: PoS, perception of the self; PF, planned future; SC, social competence; PS, personal structure; FC, family coherence; SS, social support; RSA, resilience Scale for Adults; M, mean; SD, standard deviation.

For the total scale, a significant difference in resilience values according to education level was found, with those with a higher degree (Doctorate and Master) presenting a higher resilience than those with a Bachelor’s degree (*P* < .034). Also, a significant difference in total resilience was found between occupations, with nurses presenting the lowest levels and medical doctors/psychologists the highest (*P* = .018). Differences between occupations were also found in PoS, PF and FC subscales (*p*_PoS_ = 0.007; *p*_PF_ = 0.008; *p*_FC_ = 011), all with nurses presenting the lowest resilience results. In PS subscale, resilience has significant differences according to education level, with those of higher education schools presenting higher resilience (*p*_FC_ = 0.002).

Table 3 shows the correlation between RSA scale and subscales and age, years of working as a healthcare professional, number of working hours per week and how they perceived their health during the COVID-19 pandemic. Although lower, a positive correlation between PoS and age, years of working (yw) and perceived health during the pandemic period was found (*r*_age_ = .205, *P* < .001; *r*_yw_ = .171, *P* = .005; *r*_health_ = .213, *P* < .001, respectively). A positive correlation between how participants rate their overall health during the COVID-19 pandemic and PF, SC, and PS subscales and total RSA scale were found (*r*_PF_ = .250, *P* < .001; *r*_SC_ = .147, *P* = .016; *r*_PS_ = .162, *P* < .007, *r*_RSA_ = .200, *P* < .001). Values in bold indicate significant *P* = 0.05.

**Table 3.** Association between RSA scale and subscales values and age, years of working, number of working hours per week and perception on the overall health (ranging from 1 to 10) during Covid-19.

	PoS		PF		SC		PS		FC		SS		Total RSA	
	*r*	*P*	*R*	*P*	*r*	*P*	*r*	*P*	*r*	*P*	*r*	*P*	*r*	*P*
Age	0.205	<**.001**	0.007	.914	–0.002	.974	0.015	.806	0.050	.408	0.038	.532	0.080	.188
Years of work in healthcare	0.171	**.005**	0.016	.790	0.014	.824	0.032	.601	0.036	.552	0.008	.891	0.069	.260
Number of working hours per week	–0.068	.269	–0.055	.375	0.011	.860	0.014	.818	–0.039	.527	0.001	.981	–0.033	.587
Perception on the overall health during the COVID-19 pandemic	0.213	<**.001**	0.0250	<**.001**	0.147	**.016**	0.162	**.007**	0.032	.597	0.093	.126	0.200	<**.001**

Abbreviations: PoS, perception of the self; PF, planned future; SC, social competence; PS, personal structure; C, family coherence; SS, social support; RSA, resilience scale for adults; r, Pearson’s correlation.

### Qualitative phase

Table 4 shows the sociodemographic characteristics of the 10 participants that were interviewed. Among them, 6 were women and 4 were men. The minimum and maximum age of the participants was 27 and 70 respectively. Most of them was working in a public hospital and with an average length of professional experience of 18.5 years.

**Table 4.** Sociodemographic characteristics of the participants in the interviews.

Participant	RSA	Gender	Age (y)	Occupation	Academic degree	Type of institution	Professional experience (y)	Leadership role
1	129	Female	34	Nurse	Bachelor’s degree	Private hospital	4	No
2	135	Female	32	Doctor	Master’s degree	Public hospital	7	No
3	141	Male	27	AHP (OT)	Master’s degree	Public hospital	6	No
4	145	Female	44	Psychologist	Bachelor’s degree	Both	20	No
5	149	Male	42	Nurse	Bachelor’s degree	Public hospital	19	No
6	200	Male	70	Psychologist	PhD	Public hospital	36	Yes
7	204	Female	29	Nurse	Bachelor’s degree	Public hospital	6	No
8	219	Female	44	Psychologist	PhD	Medical emergency	21	Yes
9	221	Male	62	AHP (Rad)	Bachelor’s degree	Public hospital	33	No
10	222	Female	60	Psychologist	Bachelor’s degree	Both	33	No

Abbreviations: AHP, allied health professional; OT, occupational therapist; Rad, radiologist; Ph.D., doctorate degree; RSA, resilience scale for adults (ranging from 33 to 231).

**Figure 1.**
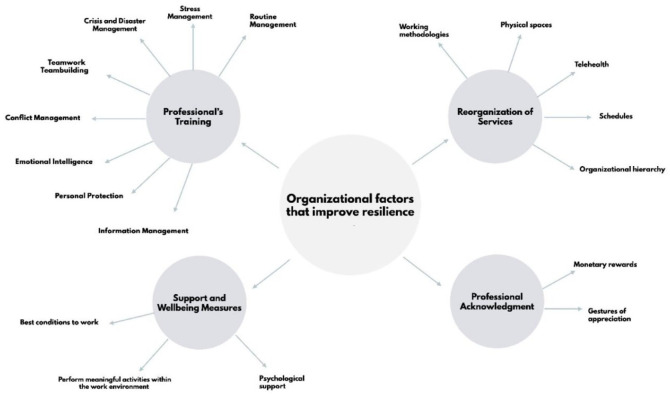
Organizational factors that improve individual resilience.

Through the analysis of the interviews, 4 categories emerged that intend to reflect the perspectives of healthcare workers in Portugal regarding organizational factors that improve individual resilience: “Professional’s Training,” “Support and Wellbeing Measures,” “Reorganization of Services” and “Professional Acknowledgment” (Figure 1).

### Professional’s training

Professional’s training was a theme addressed by all participants. They considered that training is fundamental for changing practices and attitudes that can influence resilience. Participants highlight that the more information and tools they have, the better the care provided will be “*because (. . .) the more informed we are, the better we’ll be, right*?” (P1) and the better a crisis will be experienced in the future, such as a new pandemic “*training people to be able to deal better, to be able to face it is an important strategy for overcoming a general crisis, right*?” (P8). The training could be focus on general competences, namely crisis and disaster management, but also in specific personal skills such as stress management. The areas of training highlighted by the participants are shown in Table 5.

**Table 5.** Proposed training subjects.

Subject	Quotes
Stress management	*“Having trainings (. . .) to know how to manage stress, to know how to manage when things go less well, I think essentially it’s very much that way.” (P2)* *“An office that would give us trainings on having weapons to deal with these stressful situations” (P5)* *“All those ways of dealing with situation should also be taught (. . .) some workshops, some things like that to deal with stressful situations” (P1)*
Crisis and disaster management	*“I think there are really trainings for dealing with disasters and those things the public health doctors are very connected to that area. I think it was important not just the public health doctors to do that, but we all the professionals in the hospitals are not all doctors.” (P2)* *“Training like this for everyone [crisis and catastrophe] [. . .], because it teaches us precisely how to act in the face of something that no one is expecting.” (P4)*
Teamwork/teambuilding	*“I think the main thing is really to develop those skills of working as a team.” (P2)* *“So it is important that training is also done in leadership in the organization and management of health services as well” (P6)* *“Team spirit, perhaps there should be training in that sense also to unite the team and have activities, it’s something that I already did from time to time in the hospital, activities with everyone” (P3)*
Conflict management	*“Conflict management in a normal context I think is one thing, conflict management in a pandemic situation is a result of another aspect, the basis is not the same. The teaching of strategies to regulate ourselves, I think it is very important” (P10)*
Emotional intelligence	*“I think there was a lack of an emotional management, I’m not sure how it’s done, but at least I think that everyone who belonged to the teams should have the chance to talk about what happened, (. . .) talk about what happened and know how it influenced our lives.” (P8)* *“I think on a more individual level, strategies of adaptation and emotional intelligence strategy” (P10)*
Personal protection	*“The trainings could be at the level of infection protection, infection control.” (P9)* *“At the level of equipment, of personal protective equipment, I think it is also very important ah to understand or have information on how to use them” (7)*
Information management	*“The access to information, but that we need to know how to manage that information, so ah an important training that targets these skills” (P8)* *“I think it was important to do the workshop on the information itself to professionals about what is pandemic. We have access to a lot of information, but maybe filter a little bit what is important for each, for each professional category.” (P2)*
Routine management	*“I think it was important to have training either from psychologists or occupational therapists because of the routines, the importance of routines. [. . .] importance of our, of the structuring of our routines and the importance that we, that we give to the activities. I think it was important.” (P3)*

### Support and wellbeing measures

Following professional training, participants consider that alongside the training, there should be a focus on supporting the well-being of healthcare professionals. This support can focus on measures to promote employees’ well-being or the delivery of specific psychological support. The participants of this study considered that health services benefited from having healthcare professionals more satisfied with working conditions and more professionally and personally fulfilled. Thus, the participants, based on the support they felt was lacking during the pandemic, identified some support and well-being measures that they considered could be useful for professionals and consequently would influence care delivery “*If the professionals, those who are exactly in the field are given the best conditions to work, at the bottom of the chain, in the pyramid, everybody wins*” (P7); “*I think the more, and it’s scientifically proven, that the more time we give people to do what makes us happy, the more productive people are going to be*” (P1).

The participants consider that health institutions should allow health professionals to have time to perform meaningful activities within the work environment to increase their well-being “*organize themselves somehow for us to have some time when we could do something, some kind of activity where we could relax, even if it was just for* 15* minutes*.” (P3)

Outside of the work environment, but related to the demands of that same environment, the participant considered that they should also be able to enjoy more time for meaningful activities, “*If he/she then has no time to be with his family, to be with himself, to do absolutely nothing*” (P4), it is difficult for him/her to feel well.

Moreover, they also consider that there is a gap in the psychological support provided to health professionals, either by the absence or by the inability to respond to all the needs that should be addressed, since they consider that it would bring many benefits for the mental health of professionals, “*I think that psychological support is fundamental, [. . .] we have an office, but I mean, it’s not enough for the whole hospital, one person for a whole hospital is impossible*” (P2).

### Reorganization of services

All participants mentioned that, in order to really achieve changes that are impactful and sustainable, there is a need to promote a restructuring of the services, without which it will be very difficult to implement the other measures identified. Those measures should include changing working methodologies and schedules, making physical spaces more functional, implementing telehealth and other technological innovations, and improving organizational hierarchy. Participants consider that there is a need for an adjustment in the organization of health services to improve working conditions for professionals, “*We don’t have the working conditions. It’s far short of what is needed, what was needed, what would be needed*” (P9). From this, the following restructurings arise:

- Working methodologies: “*I think it is important in Portugal to have formalization of work methodologies and I think this has failed miserably*” (P4); “*In terms of service organization I think we lack protocols*” (P1).
- Physical spaces: “*Having availability of places* for example *for group interventions*” (P10); “*I think that it only occurred to me the issue of beds and the number of vacancies that are needed [. . .]so that there wouldn’t have to be all this confusion with patient transfers and insufficient numbers of beds*” (P5).
- Telehealth: “*I think that telemedicine has helped a lot”* (P1); *"Health professionals (. . .) were realizing that there are certain things they can do remotely.”* (P5).
- Schedules: “*I think that working shifts is not beneficial, although there is this need it ends up not, not, not being beneficial*" (P1); “*The excessive load that we are daily is big and so we had to decrease the load*” (P5).
- Organizational hierarchy: “*There should be organizational charts of priorities at the level of health professionals*” (P2); “*The leaderships are very closed*” (P7).

### Professional acknowledgment

The participants’ self-perception of a devaluation of their social role was almost universal, and particularly so during the pandemic period. It is their opinion that, as healthcare professionals, they play a primary role in society and are constantly exposed to risks and situations of pressure and stress. These issues during the COVID-19 pandemic were even more evident, however, the participants consider that neither during this phase nor before had sufficient recognition and appreciation of their work - *“I don’t think these people are ever going to be valued enough. They will never be given the appreciation and I don’t mean the gratification, but at least the appreciation of what they did that I don’t think anyone is very aware of*” (P5); “*We went through complicated times, not tearing others apart, not tearing other professions apart, but I think that valuing our work more was perhaps the most appropriate measure* (P3).

According to the participants, this acknowledgment can be done in several ways and one of them is monetary rewards - “*The support that should have been done and that should have been taken advantage of this opportunity to receive was an effectively remunerative suppor*t” (P1).

Similarly, participants indicate that comforting expressions and gestures of appreciation are always gratifying to be received - “*It always feels good to hear a thank you from a manager, doesn’t i*t?” (P7).

Thus, the participants show the importance that self-perception of recognition and appreciation as professionals and people has in contributing to the promotion of resilience.

## Discussion

This study examined the resilience levels of healthcare professionals in Portugal and followed up with a qualitative study using in-depth interviews to understand their perspectives regarding organizational factors that improve individual resilience.

First, the results evidenced that the sample resilience level was moderate. Some international studies assessed healthcare professionals’ resilience and their levels were moderate to high.^26-28^ Nevertheless, there is a diversity of experiences, practice roles and care routines among health professionals that results in different levels of resilience. According to the results of this study, nurses had lower resilience levels in opposition to psychologists and physicians, which had the highest RSA score. Several studies^29,30^ conducted with nurses showed also lower levels of resilience compared to other occupations. This may happen, since these professionals were in direct contact with patients and perform several functions, such as administering medication, performing vaccinations, performing physical examinations, and heavier workload due to working in shifts. In addition, nurses assumed a more active role in the COVID-19 pandemic, compared to other healthcare professionals, and their occupational balance was altered.^31^ Also, the COVID-19 pandemic made nurses constantly fear contracting the virus and infecting their families,^30^ a distress that is higher for healthcare workers in emergency services.^32^

Psychologists are expected to have extensive knowledge in several areas associated with mental health, namely in the use of adaptive coping strategies that allow managing emotions in a positive and meaningful way,^33^ as this study confirmed. The moderate levels of resilience among physicians were not expected, which, according to the literature, is one of the professional groups with lower resilience, with a burnout prevalence ranging from 0% to 80.5%^34^ and other mental health problems such as depression and anxiety.^35^ These problems were especially prevalent during the COVID-19 pandemic.^36^ Despite the moderate levels of resilience presented by the physicians in this sample, the authors verified, that one of the worst values of resilience was from a female physician. During her interview it was possible to observe that the high levels of anxiety and fatigue complaints were due to a lack of professional support from a supervisor, high workloads, with many hours without breaks; complaints that were supported by the literature. According to a systematic review and meta synthesis of qualitative studies, healthcare professionals’ resilience stems mostly from their professional identity, collegial support, effective communication from supportive supervisors, and the flexibility to engage in self-care and growth experiences.^37^ The literature outlines that the characteristics that promote high levels of resilience are self-efficacy, optimism, and adaptive coping strategies.^38^ Healthcare professionals with a high level of self-efficacy are more likely to implement effective strategies to deal with certain challenges, taking measures to cope with adversities.^39,40^

Nevertheless, the organizational factors have a higher impact on the promotion of individual resilience as well. Through our interviews, the participants highlight the importance of professional training, measures to increase support and well-being, the need to reorganize services, and professional acknowledgment. Moreover, the literature suggests the implementation of resilience-oriented interventions through education and training to strengthen healthcare workers’ defenses against various mental and psychological consequences of the pandemic^12,17^ The training subjects are diversified which highlights the need for great variety in terms of the knowledge and skills required of professionals, since training is a predictor of professionals’ readiness for conducting advance care planning in terms of perceived relevancy, willingness, and confidence.^41^ Providing information through videos and pamphlets has been shown to be an effective strategy for managing stress in health professionals.^42^ Similarly, training of healthcare professionals on this topic could be delivered using digital tools and approaches such as mindfulness, promotion of self-care, psychological counseling, information from the different platforms and organizational support services.^43,44^

Under the circumstances of a pandemic, appropriate measures should be established to ensure the well-being of healthcare professionals in the work environment. Some support and well-being measures were identified as an inevitability, which is corroborated by other studies that also point to the strengthening of psychological care services for teams of healthcare professionals as an important measure, since healthcare professionals’ need more mental support from superiors and services.^45^ The participants confirmed the importance of promoting balanced and positive routines for healthcare professionals, such as days off allowing that the professionals can engage in self-care activities and have healthy lifestyles, such as maintaining proper nutrition, a good sleep routine, exercising, and establishing social connection.^46^ In addition, social support is crucial for individuals’ well-being and mental health and the evidence reports that the existence of a wider social network is associated with greater resilience.^47^ Furthermore, several studies have shown that social support, mainly from family and friends, has a positive relationship with the mental health of healthcare professionals, especially during traumatic and stressful events.^48,49^ Subsequently, establishing social contacts, even if in a different way than usual (eg, through online communication) was essential to promote healthy emotional states in healthcare professionals. Some of these topics are part of sub-scales that make up the RSA and may explain the differences found.

Services reorganization was also mentioned by several participants. Working methodologies, physical spaces, schedules, organizational hierarchy, and information storage were emphasized. The COVID-19 pandemic promoted the adoption and development of organizational innovations that could improve access to primary healthcare by removing, at least temporarily, certain barriers.^50^ It is essential that leaders and managers of healthcare services use this learning experience to improve services organization.

Moreover, professional acknowledgment was an aspect that emerged in the participants’ discourse revealing some dissatisfaction with their profession, both in terms of remuneration and appreciation. Regarding dissatisfaction, recent studies showed that the main reasons for dissatisfaction were excessive working hours, insufficient remuneration, too much administrative workload and few opportunities for promotion .^51,52^ Studies report the need to provide economic incentives to professionals during health crises, ensuring their motivation.^53^ On the other side, working in a high-quality facility increases worker satisfaction and willingness to remain in the profession.^54^ Hence, evidence suggests that, for instance, a high level of satisfaction with the work has a significant impact on providing better patient care as well as reducing the risk of professional burnout of nurses.^55^

Concerning appreciation, this emphasis on the importance of public recognition of the work of healthcare professionals has also been highlighted in the literature. Shan et al argue that positive public responses, such as encouragement, acknowledgment, and material donations, boosted the work engagement and well-being of healthcare professionals.^56^ Another study found out that workplace civility and gratitude have a significant impact on job satisfaction and patient safety.^57^

This study is not without limitations. The study was conducted many months after the coronavirus pandemic onset, and this possibly affected not only the participants’ ability to proper recall those specific times, but also the reliance on self-reported data. Additionally, the study’s findings may not be generalizable to all healthcare professionals in Portugal, as the sample may not be fully representative of the entire population. However, the current study has shed light on the importance of addressing organizational factors to improve individual resilience.

Further studies exploring resilience levels and associated factors among healthcare professionals in different countries or regions are required. Additionally, comparing resilience levels across different healthcare systems, cultural contexts, and socio-economic backgrounds can provide a broader perspective on the role of organizational factors in shaping resilience among healthcare professionals.

## Conclusions

This mixed-method study aimed to assess the overall resilience levels among healthcare professionals in Portugal and gain insights into their perspectives on organizational factors that enhance individual resilience. Through the quantitative was found that the level of resilience was moderate, with the lowest levels manifesting in nurses. Subsequently, a qualitative phase led to the emergence of 4 major themes which were highlighted as responsible for greater resilience: “Professional’s Training,” “Support and Wellbeing Measures,” “Reorganization of Services,” and “Professional Acknowledgment.” Some of the mechanisms used by healthcare personnel to boost their resilience and deal with the pandemic were looking for psychological and family support, reorganize personal and work routines, seek for professional training in different areas such as crisis and stress management, and taking care of themselves using personal protection.

The findings of this study hold significant implications for the development of targeted interventions and support systems aimed at enhancing resilience and promoting well-being among healthcare professionals. By improving resilience levels, these professionals are more likely to experience positive effects on their mental health, job satisfaction, and on the overall quality of healthcare provided to patients. It is crucial for organizations and policymakers to consider these findings when designing strategies to support healthcare workers, not only in times of crisis but for the smooth running of health services on a daily and continuous process.

## Supplemental Material

sj-docx-1-his-10.1177_11786329231198991 – Supplemental material for Healthcare Professionals’ Resilience During the COVID-19 and Organizational Factors That Improve Individual Resilience: A Mixed-Method Study Click here for additional data file. Supplemental material, sj-docx-1-his-10.1177_11786329231198991 for Healthcare Professionals’ Resilience During the COVID-19 and Organizational Factors That Improve Individual Resilience: A Mixed-Method Study by Raquel Simões de Almeida, Ana Costa, Inês Teixeira, Maria João Trigueiro, Artemisa Rocha Dores and António Marques in Health Services Insights
