# Corrigendum to “Healthcare Professionals’ Resilience during the COVID-19 and Organizational Factors That Improve Individual Resilience: A Mixed-Method Study”

**DOI:** 10.1177/11786329241298050

**Published:** 2024-11-04

Simões de Almeida R, Costa A, Teixeira I, Trigueiro MJ, Dores AR, Marques A. Healthcare Professionals’ Resilience During the COVID-19 and Organizational Factors That Improve Individual Resilience: A Mixed-Method Study. *Health Services Insights.* 2023;16. doi:10.1177/11786329231198991

Funding section has been updated and correct funding section is as below:

Funding

The author(s) disclosed receipt of the following financial support for the research, authorship, and/or publication of this article: This work was supported by Fundação para a Ciência e Tecnologia (FCT) through R&D Units funding (UIDB/05210/2020). Funded by the European Union (Grant Agreement ERASMUS+ 2021-2-AT01-KA220-VET-0000049921). Views and opinions expressed are however those of the author(s) only and do not necessarily reflect those of the European Union or OeAD-GmbH. Neither the European Union nor the granting authority can be held responsible for them.

This work was also supported by Fundação para a Ciência e Tecnologia (FCT) through R&D Units funding (UIDB/05210/2020).

The article is updated online.

