# Supplementary material for: Healthcare Professionals’ Resilience During the COVID-19 and Organizational Factors That Improve Individual Resilience: A Mixed-Method Study
Source: Health Serv Insights. 2023 Sep 19;16:11786329231198991. doi: 10.1177/11786329231198991 (PMC10510343; doi:10.1177/11786329231198991)
Supplement: sj-docx-1-his-10.1177_11786329231198991 – Supplemental material for Healthcare Professionals’ Resilience During the COVID-19 and Organizational Factors That Improve Individual Resilience: A Mixed-Method Study [file sj-docx-1-his-10.1177_11786329231198991.docx]

Interview Guide

1. General Questions about function and responsibilities

-  What is your function and what are your responsibilities in your organization?

-  Since when are you in this position?

2. Question experiences start phase of CoVid19

- Please remember back to the start of the pandemic CoVid19 crisis in 2020. Can you please give me a brief description of how you experienced the pandemic personally at your workplace.

3. Questions regarding working routines and challenges of CoVid19 in 2020 / 2021

-  With which challenges you were confronted at the start phase of CoVid19?

-  Which ones were the most challenging ones you were faced during this time and how did you deal with them?

-  How did your daily work routine changed due to the CoVid19 regulations and how did you to adjust your daily working routines during the period 2020/2021 of the pandemic? What worked well and what worked not so well?

-  Are you aware of any good practice in that context? What would have been most helpful from your point of view?

4. Questions regarding patient care during CoVid19 in 2020 / 2021

-  What were the most serious concerns of your patients between 2020 and 2021 of the pandemic?

-  What were your biggest concerns whilst dealing with patients?

5. Questions related to organizational support.

- In your opinion, how has your organization coped with the pandemic so

-  Which kind of support did you get from your organization during the start phase of CoVid19? Could you please specify? Was the support by your organization helpful to you and in which way?

-  Which kind of further support would you have wished for by your organization?

-  Which kind of support did you get from your directly assigned supervisor / leader during the start phase of CoVid19? Could you please specify? Was the support by her/him helpful to you and in which way?

-  Which kind of further support would you have wished for by your supervisor / leader?

6. Questions related to individual skills and stress.

-  To what extent and how were your experiences before the pandemic useful to cope with the challenges brought by the pandemic?

-  According to you, what are the most important skills for persons in your professional role in general to cope with challenging situations due to a pandemic?

-  Which one of those skills did you make use of to cope with the challenges you described earlier?

-  What were your biggest sources of stress for you during the pandemic?

-  Which kind of measures did you take to reduce the stress?

-  Which skills do you think you would be needed in getting trained to deal with this type of situations in the future?
